# Meta-analysis of associations between *TCF7L2* polymorphisms and risk of type 2 diabetes mellitus in the Chinese population

**DOI:** 10.1186/1471-2350-14-8

**Published:** 2013-01-12

**Authors:** Jinjin Wang, Fulan Hu, Tianping Feng, Jingzhi Zhao, Lei Yin, Linlin Li, Yan Wang, Qian Wang, Dongsheng Hu

**Affiliations:** 1 Department of Epidemiology, College of Public Health, Zhengzhou University, Zhengzhou, 450000, People’s Republic of China; 2 Department of Epidemiology, Public Health College, Harbin Medical University, Harbin, 150086, People’s Republic of China; 3 Military Hospital of Henan Province, Zhengzhou, 450000, People’s Republic of China; 4 Shenzhen University School of Medicine, Shenzhen, 518000, People’s Republic of China

**Keywords:** Type 2 diabetes, T2DM, TCF7L2, SNPs, Meta-analysis

## Abstract

**Background:**

Associations between transcription factor 7-like 2 (*TCF7L2*) polymorphisms and type 2 diabetes mellitus (T2DM) have been evaluated extensively in multiple ethnic groups. *TCF7L2* has emerged as the strongest T2DM susceptibility gene in Europeans, but the findings have been inconsistent in the Chinese population. The purpose of this meta-analysis was to evaluate the associations between *TCF7L2* single nucleotide polymorphisms (SNPs) and T2DM risk in the Chinese population.

**Methods:**

We performed searches in the PubMed, EMBASE, Cochrane, and Chinese databases (CNKI, CQVIP and Wanfang databases) for literature published from January 2007 to February 2012. We reviewed all relevant articles on *TCF7L2* polymorphisms and susceptibility to T2DM in the Chinese population written in English and Chinese. Two reviewers extracted data independently using a standardized protocol, and any discrepancies were adjudicated by a third reviewer. Fixed-effects and random-effects meta-analyses were performed to pool the odds ratios (ORs). Publication bias and heterogeneity were examined.

**Results:**

A total of 21 articles were confirmed to be eligible for and included in this meta-analysis: 7 (with 3942 cases and 3502 controls) concerning rs11196218 (IVS−/+4G>A), 8 (with 3377 cases and 2975 controls) concerning rs290487 (IVS3−/+C>T), and 14 (with 7902 cases and 7436 controls) concerning rs7903146 (IVS3−/+C>T). Overall, the results showed a significant association between rs7903146 and T2DM risk. The pooled ORs were 1.54 for the comparison of T and C alleles (95% CI [confidence interval]: 1.37–1.74, *p* = 1.47 × 10^-12^, *I*^2^ = 25.20%) and 1.56 for TC heterozygotes and CC homozygotes (95% CI : 1.38–1.76, *p* = 8.25 × 10^-9^, *I*^2^ = 21.00%). The rs11196218(IVS4G>A) and rs290487 (IVS3C>T) SNPs were not associated with T2DM risk.

**Conclusions:**

The rs7903146 SNP of the *TCF7L2* gene is associated with increased susceptibility to T2DM in the Chinese population as a whole as well as northern Chinese and southern Chinese as subgroups.

## Background

Type 2 diabetes mellitus (T2DM) is the most common form of diabetes, accounting for 90% of cases in the world [1]. The incidence and prevalence of T2DM has reached epidemic proportions worldwide. It is estimated that China will contribute almost 38 million patients to the global burden of diabetes by 2025 [2,3]. Moreover, the incidence of T2DM in children has increased dramatically in the past decades [4]. Although T2DM pathogenesis remains to be fully elucidated, genetic susceptibility appears to play a crucial role in the etiology and manifestation of the disease [5-7]. Notably, single nucleotide polymorphisms (SNPs) of the transcription factor 7-like 2 (*TCF7L2*) gene have been reported to affect T2DM susceptibility by indirectly altering expression of GLP-1 [8], which in addition to insulin, plays a critical role in blood glucose homeostasis [9].

In recent years, numerous studies examining various ethnic groups have demonstrated an association between *TCF7L2* genotype and T2DM. However, the results have been inconsistent [10-30]. Five meta-analyses of the association between *TCF7L2* SNPs and T2DM have been published [31-35]. The first published in 2007, demonstrated a significant association between the rs7903146 SNP and T2DM in Moroccans (allelic OR = 1.56, 95% CI [confidence interval]: 1.29 – 1.89, *p* = 2.9 × 10^-6^) and Austrians (allelic OR = 1.52, 95% CI: 1.29–1.78, *p* = 3.0 × 10^-7^) [33]. The second study published in 2009, demonstrated significant associations between four *TCF7L2* SNPs (rs7903146, rs12255372, rs7901695 and rs11196205) and T2DM [32]; however, only two articles examining Chinese subjects were included in that meta-analysis. The third meta-analysis, also published in 2009 but which included six studies of Chinese population samples, concluded that there were five *TCF7L2* SNPs (rs7903146, rs12255372, rs11196205, rs290487 and rs11196218) associated with T2DM risk among East-Asian people [31]. The fourth study, also published in 2009, focused on differences in the association between rs7903146 and T2DM among different inter-East Asian populations; three of the six studies had examined Chinese population samples [34]. The fifth study was a three-stage analysis designed to identify susceptibility loci for T2DM among East Asians. In the first stage, the researchers performed a meta-analysis of eight T2DM genome-wide association studies (6952 cases and 11,865 controls) for established T2DM risk loci and confirmed a significant association between rs7903146 and T2DM risk among East-Asians (OR = 1.16, 95% CI: 1.02*–*1.31, *p* = 2.5 × 10^-2^) [35]; however, none of those eight studies examined Chinese people.

Sixteen studies [14,15,18-30] examining this question in Chinese population samples have been published since the aforementioned meta-analyses. However, their results have been inconsistent. Thus, the potential association between *TCF7L2* polymorphisms and T2DM in Chinese population remains inconclusive. In this study, we performed an up-to-date meta-analysis to assess the contribution of *TCF7L2* polymorphisms to T2DM susceptibility specifically in Chinese people.

## Methods

### Primary search strategy

We conducted a search of the PubMed, EMBASE, Cochrane, and Chinese literature databases (CNKI, CQVIP, and Wanfang databases) for articles published from January 2007 to February 2012. The following subject terms were used: ‘*TCF7L2*’, ‘genetic polymorphism’, ‘Chinese’, and ‘T2D/T2DM’. Only human studies published in English or Chinese were retrieved for consideration. The reference lists of the selected articles were searched to identify any additional relevant articles.

### Inclusion criteria and data extraction

Studies were selected for our analysis if they reported the genotype or allele frequencies of *TCF7L2* polymorphisms in Chinese T2D patients and controls. Data were extracted by two reviewers (J. Wang and F. Hu) using a standardized data extraction form. Discrepancies were adjudicated by the third reviewer (L. Li) until a consensus was achieved on each item. A total of 21 eligible articles were included in the meta-analysis.

### Statistical analysis

The Metan module in the STATA 11.0 software package was used to perform the meta-analysis on genotype frequencies. The strength of the association between each SNP and T2DM risk was measured by determining ORs with 95% CIs. The Z test was used to determine the statistical significance of pooled ORs. Heterogeneity index (*I*^2^, 0–100) was applied to assess heterogeneity among the studies [36]. In accordance with the Cochrane reviewer’s handbook 4.2.2 [37], we accepted a high degree of heterogeneity for study pairs with an *I*^2^>50%. When heterogeneity was not an issue, the fixed effects model was applied with the Mantel-Haenszel method [38]. Otherwise, a random effects model was applied using the DerSimonian and Laird method to calculate the combined OR [39]. Subgroup analyses were performed by region (South or North) for each SNP.

Non-risk and risk alleles were represented by A1 and A2, respectively. The OR for the risk allele A2, A1/A2 was compared to that for A1/A1, and the OR for A2/A2 was compared to for A1/A1.

The present meta-analysis evaluated the association between three SNPs in *TCF7L2* gene and T2D, and the Bonferroni method [40] was employed to adjust for Type I errors. Specifically, the Bonferroni significance threshold was set at α = 0.05 / 3 = 0.017 for the 3 SNPs.

Publication bias was investigated with funnel plots [41]. Furthermore, Egger’s regression approach was adopted [42]. Power calculation was performed using PASS ( Power analysis and sample size, by Jerry) software. Population-attributable risk (PAR) was calculated based on the estimated ORs and risk allele frequencies of those SNPs that showed significant association with T2DM risk.

## Results

### Inclusion of SNPs for analysis

**Figure 1:**
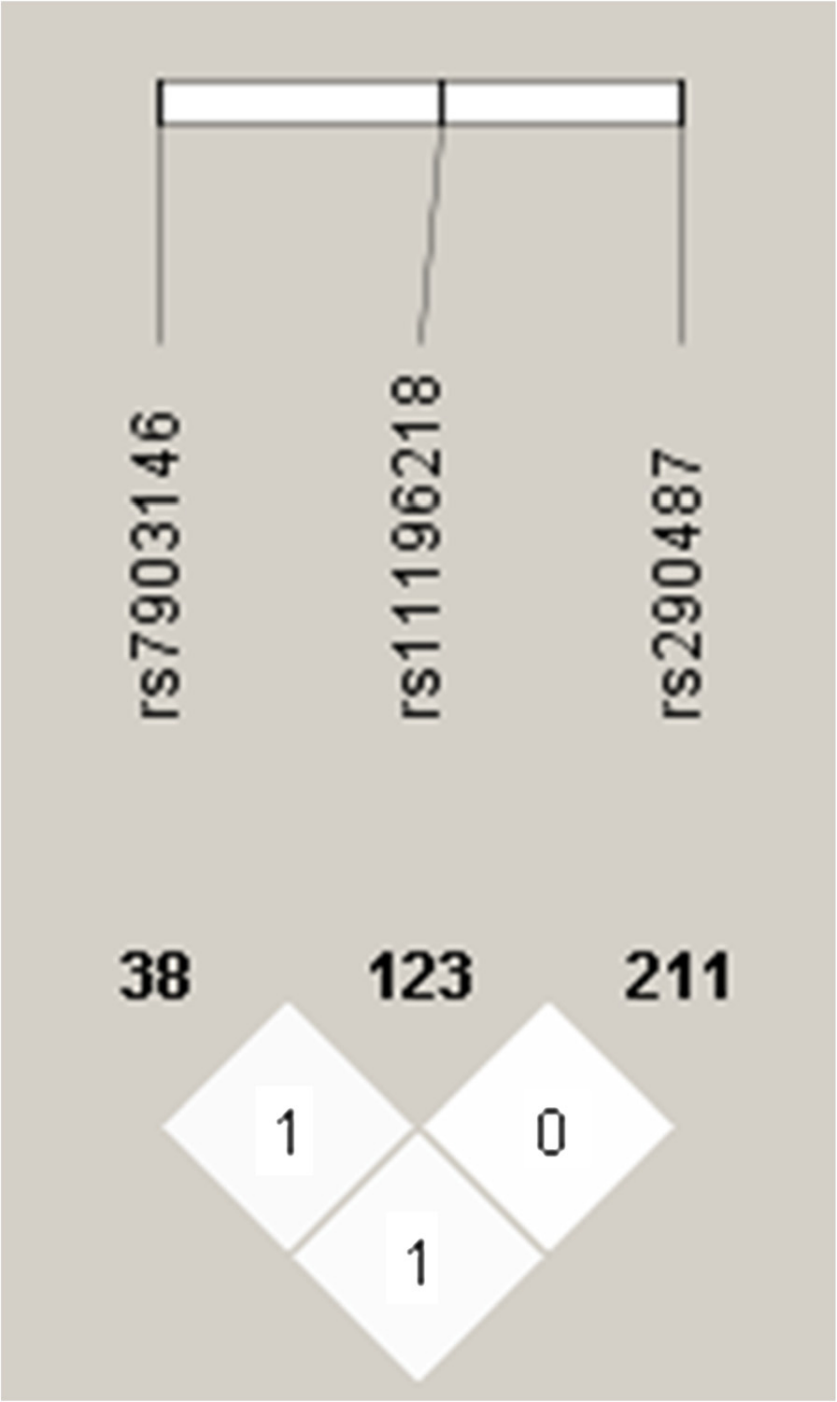
**LD plots for rs7903146, rs11196218, and rs290487 in the Chinese population.** Based on Hapmap data (HapMap Data Rel 27 PhaseII+III, Feb09, on NCBI B36 assembly, dbSNP b126).

Among SNPs previously associated with T2DM risk in the Chinese population, only three were adequately represented in the selected literature and had sufficient low minor allele frequency (MAF) to be included in our meta-analysis: rs7903146, rs11196218, and rs290487. We found that Hapmap data (HapMap Data Rel 27 Phase II+III, Feb09, on NCBI B36 assembly, dbSNP b126) indicate that, in the Chinese population, the linked disequilibrium (LD) structure places D’ at 1.0 unit between rs7903146 and rs290487, 1.0 unit between rs7903146 and rs11196218, and 0.059 unit between rs290487 and rs11196218 (Figure 1). Between rs7903146 and rs11196218, r^2^ = 0.016, between rs7903146 and rs290487, r^2^ =0.013, and between rs11196218 and rs290487, r^2^ = 0.001 (see Additional file 1: Table S1). The LD was relatively low among three SNPs in the Chinese population, so we decided to include these SNPs in our meta-analysis.

### Characteristics of included studies

**Figure 2:**
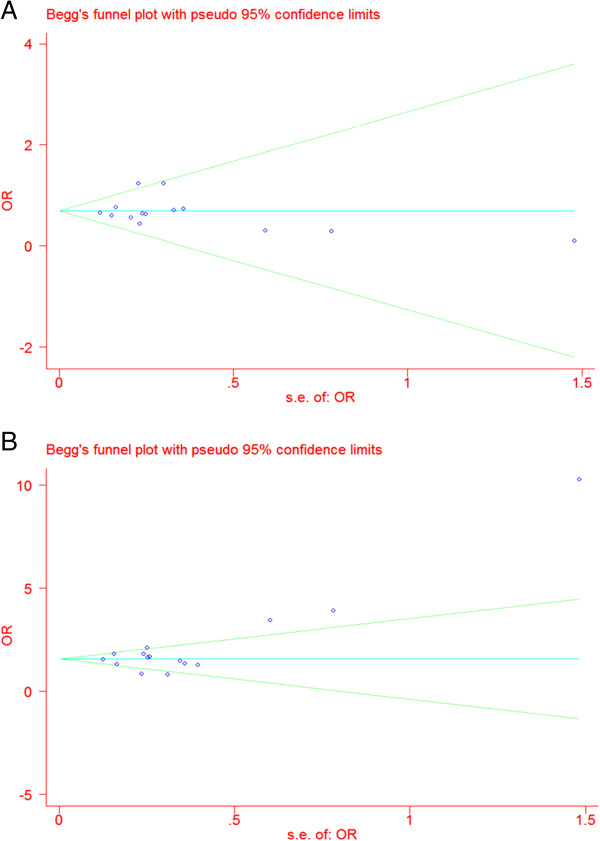
**Funnel plot for rs7903146.** Panel **A**: the ORs of allele T versus allele C of rs7903146 overall; Panel **B**: of the ORs of heterozygotes versus C homozygotes of rs7903146 overall.

Of 38 studies were identified as the potential candidates, 21 were deemed eligible for the final meta-analysis [10-30]; these 21 articles examined the three included at least one of the SNPs noted above (rs7903146, rs11196218, and rs290487). As reported in detail Additional file 1: Table S2, we examined seven studies (total of 3942 cases and 3502 controls) related to rs11196218 (ISV4−/+G>A), eight studies (total of 3377 cases and 2975 controls) related to rs290487 (ISV3−/+C>T), and fourteen studies (total of 7902 cases and 7436 controls) related to rs7903146 (ISV3−/+C>T). Our examination of heterogeneity among studies (see Additional file 1: Table S3) revealed a high heterogeneity among studies for rs11196218 and rs290487. As shown in Figure 2 and Additional file 1: Table S4, both Egger’s and Begg’s tests suggested no publication bias in the associations between rs11196218, rs290487, and rs7903146 and risk of T2DM.

### Results of meta-analysis

**Figure 3:**
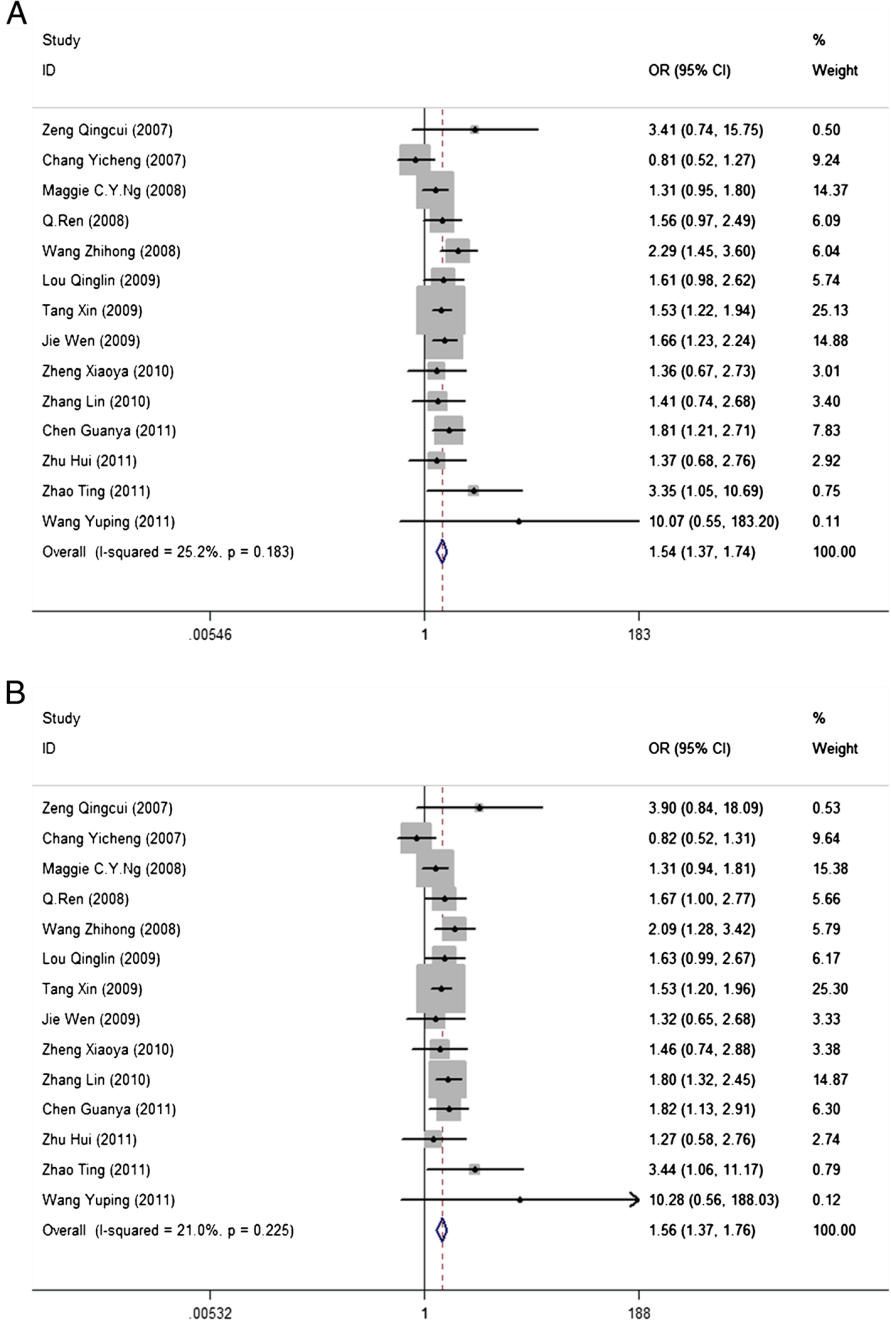
**Forest plots for rs7903146.** For the association between the T and C alleles of rs7903146 and risk of T2DM (**A**, OR = 1.54, 95% CI: 1.37–1.74, *p* = 1.47 × 10^-12^, *I*^*2*^ = 25.20%), and for the association between rs7903146 heterozygotes and C homozygotes and risk of T2DM (**B**, OR = 1.56, 95% CI: 1.34–1.76, *p* = 8.25 × 10^-9^, *I*^*2*^ = 21.00%).

The detailed results of our meta-analysis are reported in Additional file 1: Table S3. Notably, we obtained highly significant ORs for rs7903146, including the pooled OR for the T allele versus the C allele (OR = 1.54, 95% CI: 1.37*–*1.74, *p* = 1.47 × 10^-12^, *I*^*2*^ = 25.20%) and the OR for heterozygotes versus C homozygotes (OR = 1.56, 95% CI: 1.38*–*1.76, *p* = 8.25 × 10^-9^, *I*^*2*^ = 21.00%) (Figure 3). The population attributable risk (PAR) was 1.6% for the risk allele T and 2.9% for CT genotype of rs7903146. We did not find significant associations between the risk alleles for rs11196218 and rs290487 and risk for T2DM.

### Results of the subgroup analyses

When people from the northern and southern regions of China were each considered independently, we found that the allele and genotype of rs7903146 ORs remained significant for both subgroups (see Additional file 1: Table S3). In addition, we found that for rs290487 the pooled OR in subgroup analysis based on the region was 1.54 (95% CI : 1.22*–*1.94, *p* = 3.25 × 10^-5^, *I*^*2*^ = 0.00%) in the southern region of China.

### Sensitivity analysis

The sensitivity analysis was performed by omitting one study at a time. None of the individual studies influenced the final conclusions for rs290487. Likewise, removal of studies did not affect the conclusion for the risk allele A and the A homozygotes versus G homozygotes comparison for rs11196218, nor the conclusions for the risk allele T and the heterozygotes versus C homozygotes comparison for rs7903146. However, when Ying Zhang et al.’s study [24] was omitted, we found that the comparison for heterozygotes versus G homozygotes of rs11196218, which previously had a p value of 0.15, became significant (OR = 0.87, 95% CI : 0.78–0.97, *p* = 0.010, *I*^2^ = 47.80%).

## Discussions

In this study, we evaluated the effects of three well-known SNPs of the *TCF7L2* gene (rs11196218, rs290487, and rs7903146) on the susceptibility for T2DM among Chinese people by reviewing 21 eligible articles. It should be noted that these three SNPs are located in the splicing sites, and thus may affect exons and introns splicing. Our meta-analysis confirmed a significant association between the risk allele in rs7903146 (T) and risk of T2DM in the whole Chinese people regardless of region diversity. When considering the Chinese population as a whole, our data did not support significant associations for rs290487 with T2DM susceptibility, which contrasts with former results form Luo et al’s meta-analysis of studies involving East Asians [31], which showed a combined allelic odds radio for rs290487 (OR = 1.11, 95% CI : 1.03–1.19, *p* = 0.01). This difference may be due to difference in the populations considered given that the present analysis was focused on studies of Chinese people, while Japanese participants were highly represented in the former study (62.5% for rs290487). Indeed, as shown in Additional file 1: Table S5 which summarizes the MAFs for Chinese, Japanese, and European populations, MAFs are not uniform across populations (see Additional file 1: Table S5). Specifically, relative to Chinese population, Japanese population have lower MAF for rs290487 (0.384 in Japanese compared with 0.439 in mainland China). The present meta-analysis had weak power (only 9.4%, 6.1% and 9.9% power to detect associations of risk allele, CT genotype and CC genotype of rs290487) for detecting associations between rs290487 risk allele or genotype with T2DM risk (see Additional file 1: Table S6).

The significant associations between the rs7903146 SNP in *TCF7L2* and T2DM risk found in our study were similar to the findings of a large study (1529 cases and 1439 controls) by Xin Tang et al. in China [25], but contrast with some small sample studies [21,23]. It could be that the small sample studies lacked sufficient statistical power to reveal these associations.

The present results also differ from those Takeuchi et al.’s meta-analysis published in 2009 [34] that focused on the ethnic differences across inter-East Asian populations in that Takeuchi et al.’s combined OR for rs7903146 among Chinese people did not emerge as significant (OR = 1.21, 95% CI : 0.96–1.51, *p* = 0.12 ). This difference is likely due to the fact that in addition to the three Chinese studies included in their meta-analysis, 12 new papers were included in our analysis.

The results in our meta-analysis regarding the rs7903146 risk allele T and genotype CT were similar to those reported in 2009 by Tong et al. [32] who examined a variety of ethnic groups including Caucasians, North Europeans, East Asians, Indians, Africans, and others. However, our genotype TT finding differed from Tong et al.’s results. Given the ethnically broad inclusion of Tong et al’s study and the ethnically narrow inclusion of ours, there are several population-related reasons for this difference. First, the risk allele frequency in the Chinese population is much lower than that in Europeans (the minor allele frequencies was 0.024 and 0.035 in the Chinese and Japanese populations, respectively; it was 0.279 in European populations, listed in Additional file 1: Table S5. Second, there was also a substantial difference in LD structure between Chinese and European population (see Additional file 1: Table S1 for r^2^ values based on HapMap data). These differences in genetic structure between the Chinese and European populations may explain why this SNP produced different results between studies involving different ethnic groups. Due to the lower MAF of rs7903146 in Chinese populations, the estimated PARs for the T allele (1.7%) and the CT genotype (3.2%) were much lower in our analysis than the PARs reported for populations of European ancestry (17–28%) [43].

When we separated northern Chinese from southern Chinese, the pattern of our risk results changed for rs290487. Specifically, when southern Chinese subjects were analyzed separatedly, we obtained significant OR for the C homozygotes versus T homozygotes comparison for rs290487, pointing to an increased T2DM risk for C homozygotes.

Heterogeneity is a potential problem when interpreting the results of meta-analyses [44]. In our meta-analysis, high heterogeneity persisted in studies examining associations between the rs290487 the rs11196218 SNPs and T2DM risk. Because our subject pool was limited entirely to mainland Chinese persons, the ethnic heterogeneity of our study was limited. In our subgroup analysis, heterogeneity was not reduced for either of the regional subgroups. Nevertheless, gender and age likely contributed to the heterogeneity of our study population.

Violations of the Hardy-Weinberg assumptions can cause deviations from expectation due to improper sampling. The genotypes of both cases and controls did not deviate significantly from the Hardy-Weinberg equilibrium in any of the 21 reported deemed eligible for inclusion in our study.

Pooling risk estimates from multiple studies can increase statistical power. To our knowledge, our meta-analysis is the largest such study of this question selectively involving Chinese subjects. However, there are several limitations inherent in performing a meta-analysis of this kind that shoud be noted. First, due to the lower MAF of some *TCF7L2* SNPs in the Chinese population, the literature examing the relationship between these SNPs and T2DM in Chinese subjects specifically is relatively limited. Second, the sample sizes of some of the included studies were relatively small. Third, since our analysis involves previously published source data, the extent of our evalystion of potential interactions of these SNPs with T2DM is limited. Finally, given the lack of full information about gender and age of the subjects across the studies, it was not possible to perform a stratified analysis or interaction analysis based on age or gender.

## Conclusions

The rs7903146 SNP of the *TCF7L2* gene is associated with increased susceptibility to T2DM in the Chinese population as a whole as well as northern Chinese and southern Chinese as subgroups.

## Abbreviations

SNP: Single nucleotide polymorphism; TCF7L2: Transcription factor 7-like 2; CI: Confidence interval; OR: Odds ratio; BMI: Body mass index; T2DM: Type 2 diabetes; MAF: Minor allele frequency; PAR: Population attributable risk; LD: Linkage disequilibrium.

## Competing interests

The authors have no competing interests to declare.

## Authors’ contributions

DH produced the design of the study, analyzed data, and drafted the manuscript. JW contributed to the design of the study, analyzed data, and helped write the manuscript. JW, TF, JZ, LY, YW and QW conducted database searches. JW, FH, and LL conducted data extraction and statistical analyses. All authors approved the final manuscript.

## Pre-publication history

The pre-publication history for this paper can be accessed here:

http://www.biomedcentral.com/1471-2350/14/8/prepub

## Supplementary Material

Additional file 1: Table S1 LD (as r2) between TCF7L2 SNPs in this meta-analysis in Chinese v.s. Caucasians. **Table S2.** Main characteristics of the 21 studies deemed eligible for meta-analysis. **Table S3.** Results of meta-analysis for TCF7L2 polymorphisms and the T2DM susceptibility. **Table S4.** Results of Egger’s test. **Table S5.** MAFs for the three presently examined SNPs analyzed in the Chinese, Japanese, and European populations. **Table S6.** Power calculation for the present meta-analysis. Click here for file
