# Supplementary material for: Meta-analysis of associations between TCF7L2 polymorphisms and risk of type 2 diabetes mellitus in the Chinese population
Source: BMC Med Genet. 2013 Jan 12;14:8. doi: 10.1186/1471-2350-14-8 (PMC3575230; doi:10.1186/1471-2350-14-8)
Supplement: Additional file 1: Table S1 — LD (as r2) between TCF7L2 SNPs in this meta-analysis in Chinese v.s. Caucasians. Table S2. Main characteristics of the 21 studies deemed eligible for meta-analysis. Table S3. Results of meta-analysis for TCF7L2 polymorphisms and the T2DM susceptibility. Table S4. Results of Egger’s test. Table S5. MAFs for the three presently examined SNPs analyzed in the Chinese, Japanese, and European populations. Table S6. Power calculation for the present meta-analysis. [file 1471-2350-14-8-S1.doc]

**Table 1 LD (as r^2^) between *TCF7L2* SNPs in this meta-analysis in Chinese *v.s.* Caucasians**

|  | rs7903146 | rs11196218 |
| --- | --- | --- |
|  | CHB/CEU^#^ | CHB/CEU |
| rs11196218 | 0.016/0.111 |  |
| rs290487 | 0.013/0.000 | 0.001/0.007 |

* CHB: Chinese; CEU: European

Table 2 Main characteristics of the 21 studies deemed eligible for meta-analysis

| SNPs | Author | Year | Region | Genotypic distribution (case/control) | | | Allele frequencies (case/control) | | *PE* for HWE | |
| --- | --- | --- | --- | --- | --- | --- | --- | --- | --- | --- |
|  |  |  |  | GG | AG | AA | G | A | case | control |
| rs11196218 | Maggie C.Y.Ng | 2007 | Hong Kong | 264/214 | 148/171 | 21/34 | 676 (78.1%) / 599 (71.5%) | 190 (21.9%) / 239 (28.5%) | 0.96 | 0.98 |
|  | Zhang Ying | 2008 |  | 253/235 | 210/153 | 31/37 | 716 (72.5%) / 627 (73.8%) | 272 (27.5%) / 223 (26.2%) | 0.15 | 0.10 |
|  | Tang Xin | 2009 | Chengdu | 773/711 | 628/601 | 128/127 | 2174 (71.1%) / 2108 (70.3%) | 884 (28.9%) / 890 (29.7%) | 0.98 | 1.00 |
|  | Zheng Xiaoya | 2010 | Chongqing | 155/78 | 85/62 | 14/12 | 380 (74. 9%) / 218 (71.7%) | 128 (25. 1%)/ 86 (28.3%) | 0.60 | 0.95 |
|  | Ma Cong | 2010 | Shanghai | 93/67 | 123/106 | 43/31 | 307 (59.3%) / 247 (60.6%) | 211 (40.7%) / 161 (39.4%) | 0.83 | 0.3 |
|  | Zhu Hui | 2011 | Anhui | 14/10 | 127/102 | 159/188 | 156 (26.0%) / 101 (16.8%) | 444 (74.0%) / 499 (83.2%) | 0.07 | 0.39 |
|  | Qiao Huang | 2012 | Haerbin | 382/292 | 261/235 | 53/30 | 1025 (73.2%) / 819 (71.8%) | 367 (26.8%) / 295 (28.2) | 0.37 | 0.05 |
|  |  |  |  | TT | CT | CC | T | C |  |  |
| rs290487 | Chang Yicheng | 2007 | Taiwan | 258/308 | 370/352 | 132/100 | 845 (58.2%) / 968 (63.7%) | 635 (41. 8%) / 552 (36.3%) | 0.97 | 0.97 |
|  | Zhang Ying | 2008 |  | 206/165 | 252/222 | 64/76 | 664 (63.6%) / 552 (59.6%) | 380 (36.4%) / 374 (40.4%) | 0.33 | 0.93 |
|  | Q.Ren | 2008 | Beijing | 182/202 | 245/244 | 73/54 | 609 (60.9%) / 648 (64.8%) | 391 (39.1%) / 352 (35.2%) | 0.52 | 0.12 |
|  | Zhang Yong | 2009 | Jinan | 35/39 | 51/50 | 14/11 | 121 (61.5%) / 128 (64%) | 79 (38.5%) / 72 (36%) | 0.50 | 0.39 |
|  | Zou Yulian | 2009 | Kunming | 89/44 | 83/44 | 28/6 | 261 (65.3%) / 132 (71.2%) | 139 (34.7%) / 56 (29.8%) | 0.23 | 0.25 |
|  | Yu Min | 2009 | Changsha | 116/67 | 141/99 | 38/22 | 373 (63. 2%) / 233 (61.8%) | 217 (36. 8%) / 143 (38.2%) | 0.63 | 0.11 |
|  | Zhu Hui | 2011 | Anhui | 99/131 | 154/133 | 47/36 | 352 (58.7) / 395 (65.8%) | 248 (41.3%) / 205 (34.2%) | 0.31 | 0.80 |
|  | Qiao Huang | 2012 | Haerbin | 277/192 | 312/264 | 107/101 | 866 (61.9%) / 648 (56.8%) | 526 (38.1%) / 466 (43.2%) | 0.22 | 0.54 |
|  |  |  |  | CC | CT | TT | C | T |  |  |
| rs7903146 | Zeng Qingcui | 2007 | Chongqing | 110/78 | 11/2 | 0/0 | 232 (95.9%) / 158 (98.8%) | 10 (4.1%) / 2 (1.2%) | 0.60 | 0.91 |
|  | Chang Yicheng | 2007 | Taiwan | 725/717 | 35/42 | 0/1 | 1484 (97.7%) / 1476 (97.1%) | 36 (2.3%) / 44 (2.9%) | 0.52 | 0.64 |
|  | Maggie C.Y.Ng | 2008 | Hong Kong | 1394/1460 | 86/69 | 1/1 | 2873 (97%) / 2990 (97.7%) | 88 (3%) / 70 (2.3%) | 0.78 | 0.84 |
|  | Q.Ren | 2008 | Beijing | 438/463 | 41/26 | 2/2 | 917 (95.3%) / 952 (96.9%) | 45 (4.7%) / 30 (3.1%) | 0.33 | 0.02 |
|  | Wang Zhihong | 2008 | Chongqing | 371/278 | 67/24 | 8/1 | 809 (90.7%) / 580 (95.7%) | 83 (9.3%) / 26 (4.3%) | 0.02 | 0.54 |
|  | Lou Qinglin | 2009 | Jiangsu | 633/526 | 49/25 | 0/0 | 1315 (96.4%) / 1077 (97.7%) | 49 (3.6%) / 25 (2.3%) | 0.33 | 0.59 |
|  | Tang Xin | 2009 | Chengdu | 1342/1320 | 181/116 | 6/3 | 2865 (93.7%) / 2757 (95.8%) | 193 (6.3%) / 121 (4.2%) | 0.97 | 0.79 |
|  | Zheng Xiaoya | 2010 | Chongqing | 202/139 | 25/13 | 0/0 | 428 (94.27%) / 268 (95.72%) | 26 (5.73%) / 12 (4.28%) | 0.38 | 0.08 |
|  | Zhang Lin | 2010 | Changsha | 213/203 | 23/15 | 0/0 | 448 (94.9%) / 420 (96.4%) | 24 (5.1%) / 16 (3.6%) | 0.43 | 0.58 |
|  | Jie Wen | 2010 | Shanghai | 1045/1066 | 120/68 | 0/2 | 2210 (94.8%) / 2200 (96.8%) | 120 (5.2%) / 72 (3.2%) | 0.06 | 0.6 |
|  | Chen Guanya | 2011 | Enshi | 192/202 | 57/33 | 9/4 | 441 (85.5%) / 437 (91.4%) | 75 (14.5%) / 41 (8.6%) | 0.08 | 0.06 |
|  | Zhu Hui | 2011 | Anhui | 283/287 | 15/12 | 2/1 | 581 (96.8%) / 586 (97.7%) | 19 (3.2%) / 14 (2.3%) | 0.00 | 0.03 |
|  | Zhao Ting | 2011 | Qingdao | 88/110 | 11/4 | 0/0 | 184 (94.4%) / 224 (98.2%) | 11 (5.6%) / 4 (1.8%) | 0.56 | 0.85 |
|  | Wang Yupin | 2011 | Yanbian | 122/114 | 5/0 | 0/0 | 249 (98.0%) / 228 (100.0%) | 5 (2.0%) / 0 (0.0%) | 0.82 | 0.06 |

**Table 3 Results of meta-analysis for TCF7L2 polymorphisms and the T2DM susceptibility**

| SNPs | Subgroups | No. studies | A1 *v.s.* A2 | | | A1/A2 *v.s.* A1/A1 | | | A2/A2 *v.s.* A1/A1 | | |
| --- | --- | --- | --- | --- | --- | --- | --- | --- | --- | --- | --- |
|  |  |  | OR (95% CI) | *I^2^* (Cochrane *PE*) | *PE* | OR (95% CI) | *I^2^* (Cochrane *PE*) | *PE* | OR (95% CI) | *I^2^* (Cochrane *PE*) | *PE* |
| rs11196218 | overall | 7 | 0.88(0.76-1.02) | 71.30% (0.002) | 0.10 | 0.89(0.76-1.04) | 48.60% (0.07) | 0.15 | 0.84(0.66-1.07) | 34.40% (0.17) | 0.16 |
|  | region |  |  |  |  |  |  |  |  |  |  |
|  | South China | 5 | 0.82(0.67-1.01) | 76.50% (0.002) | 0.06 | 0.84(0.72-0.99) | 20.60% (0.28) | 0.03 | 0.78(0.59-1.02) | 24.50% (0.26) | 0.07 |
|  | North China | 1 | 0.99(0.83-1.19) | - | 0.53 | 0.85(0.67-1.07) | - | 0.17 | 1.35(0.84-2.17) | - | 0.21 |
|  | Not reported | 1 | 1.07(0.87-1.31) | - | 0.95 | 1.27(0.97-1.68) | - | 0.08 | 0.78(0.47-1.29) | - | 0.33 |
| rs290487 | overall | 8 | 0.98(0.80-1.19) | 85.30% (8.37×10^-8^) | 0.80 | 0.99(0.79-1.26) | 76.90% (0.06×10^-4^) | 0.96 | 1.20(0.87-1.64) | 71.90% (0.001) | 0.27 |
|  | region |  |  |  |  |  |  |  |  |  |  |
|  | South China | 4 | 0.97(0.64-1.47) | 91.60% (1.43×10^-7^) | 0.89 | 1.14(0.88-1.48) | 53.40% (0.09) | 0.31 | **1.54(1.22-1.94)** | **0.00% (0.41)** | **3.25×10^-5^** |
|  | North China | 3 | 1.03(0.80-1.33) | 75.00% (0.02) | 0.83 | 0.88(0.54-1.44) | 84.80% (0.001) | 0.62 | 1.11(0.64-1.93) | 74.40% (0.02) | 0.72 |
|  | Not reported | 1 | 0.84(0.70-1.01) | - | 0.07 | 0.91(0.69-1.19) | - | 0.49 | 0.67(0.46-1.00) | - | 0.05 |
| rs7903146 | overall | 14 | 1.54(1.37-1.74) | 25.20% (0.18) | 1.47×10^-12^ | 1.56(1.38-1.76) | 21.00% (0.23) | 8.25×10^-9^ | 1.75(0.94-3.26) | 0.00% (0.66) | 0.08 |
|  | region |  |  |  |  |  |  |  |  |  |  |
|  | South China | 11 | **1.52(1.35-1.72)** | **28.30% (0.18)** | **1.72×10^-8^** | **1.52(1.34-1.73)** | **22.20% (0.23)** | **7.63×10^-4^** | 1.85(0.96-3.56) | 0.00% (0.58) | 0.07 |
|  | North China | 3 | **1.89(1.23-2.88)** | **30.00% (0.24)** | **2.18×10^-6^** | **2.04(1.30-3.21)** | **21.50% (0.28)** | **1.98×10^-5^** | 1.06(0.15-7.54) | - | 0.96 |

A1 risk allele; A2 non-risk allele; *PE p*-value for meta-analysis;

Statistical significant results in our subgroup analyses are highlighted in red; All significant *PE* values are in bold.

**Table 4 Results of Egger’s test**

| SNPs | Genetic models | *PE* |
| --- | --- | --- |
| rs11196218 | A/G | 0.38 |
|  | AG/GG | 0.60 |
|  | AA/GG | 0.40 |
| rs290487 | C/T | 0.77 |
|  | CT/TT | 0.84 |
|  | CC/TT | 0.45 |
| rs7903146 | T/C | 0.06 |
|  | TC/CC | 0.05 |
|  | TT/CC | 0.56 |

*PE* *p*-values for publication bias from Egger’s test

Table 5 MAFs for the three presently examined SNPs analyzed in the Chinese, Japanese, and European populations

| Allele | MAF | | |
| --- | --- | --- | --- |
|  | Chinese | Japanese | European |
| rs11196218 (A) | 0.262 | 0.203 | 0.268 |
| rs290487 (C) | 0.439 | 0.384 | 0.827^*^ |
| rs7903146 (T) | 0.024 | 0.035 | 0.279 |

***** T allele is the minor allele for rs290487 in the European population

**Table 6 Power calculation for the present meta-analysis**

| SNP | Power | | |
| --- | --- | --- | --- |
|  | A1 *v.s.* A2 | A1/A2 *v.s.* A1/A1 | A2/A2 *v.s*. A1/A1 |
| rs11196218 | 98.4% | 97.1% | 97.1% |
| rs290487 | 9.4% | 6.1% | 9.9% |
| rs7903146 | 100.0% | 100.0% | 80.3% |
